# Nutritional Immunology: A Multi-Dimensional Approach

**DOI:** 10.1371/journal.ppat.1002223

**Published:** 2011-12-01

**Authors:** Fleur Ponton, Kenneth Wilson, Sheena C. Cotter, David Raubenheimer, Stephen J. Simpson

**Affiliations:** 1 School of Biological Sciences, The University of Sydney, Sydney, New South Wales, Australia; 2 Lancaster Environment Centre, Lancaster University, Lancaster, United Kingdom; 3 School of Biological Sciences, Queen's University, Belfast, United Kingdom; 4 Institute of Natural Sciences, Massey University, Auckland, New Zealand; The Fox Chase Cancer Center, United States of America

Nutrition is critical to immune defence and resistance to pathogens, with consequences that affect the health, welfare, and reproductive success of individual organisms [1], [2], and also has profound ecological and evolutionary implications [3]–[5]. In humans, under-nutrition, notably of protein, is a major contributor to morbidity and mortality due to infectious diseases, particularly in the developing world [1]. Likewise, over-nutrition and its associated metabolic disorders may impair immune function, disrupt the relationship with symbiotic and commensal microbiota, and increase susceptibility to infectious disease [6]. Despite the undoubted importance of nutrition to immune defence, the challenge remains to capture the complexity of this relationship. There are three main aspects to this complexity: (i) nutrition is a complex multi-dimensional problem for hosts, pathogens, and commensals; (ii) host immunity is a complex, multi-dimensional trait; and (iii) nutrition and immunity interact via multiple direct and indirect pathways, including involvement of the host's microbiota.

## Nutrition Is a Multi-Dimensional Problem for Hosts, Pathogens, and Commensal Organisms

Although widely used, the terms “over-nutrition” and “under-nutrition” are rarely defined in studies, and often the key nutritional variables have not been identified. Many studies consider foods as uniform commodities and manipulate the amount available without considering the food's nutritional composition or having a quantitative understanding of the animal's nutrient requirements (see for instance [7], [8]). Other studies focus on a single dietary attribute (typically its calorie content) or nutritional component (e.g., the amount of protein or nitrogen in the diet), and experimentally manipulate this whilst maintaining other dietary components at a constant level, thus confounding changes in the focal nutrient/attribute with changes in the ratio of nutrients in the diet [9]–[11]. Although these studies have had their successes, we believe that this single currency approach provides only a limited understanding of the nutritional constraints on pathogen defence (see also [2]). The Geometric Framework (GF) [12]–[14] was specifically developed to capture these multi-dimensional aspects of nutrition and offers promise for the study of nutritional immunology, allowing quantitative predictions that can be statistically tested. The GF identifies nutritional optima (intake and growth targets) in multi-dimensional nutritional space and thus provides a rigorous definition and quantification of “under”- and “over”-nutrition, as well as a mean of associating immune responses, host performance, responses of host microbial communities, and pathogen growth rates with particular nutritional states.

The importance of considering the simultaneous and interactive effects of multiple nutrients when studying immune function has been illustrated by a number of studies on insects and rodents [15]–[18]. For example, Peck et al. [18] found that mice survived better on diets containing a higher ratio of protein (P) to carbohydrate (C) following inoculation with *Salmonella typhimurium*. Similarly, the ability of caterpillars to resist viral and bacterial infection increased as dietary P:C rose, and infected insects selected a higher protein diet, indicating a form of nutritional self-medication [15]–[17].

Hosts are not the only organisms facing the complexity of nutrition. Parasites and pathogens rely on the host for provision of resources and may not share the same nutritional requirements, setting up the potential for resource competition and manipulation between the different parties [18]–[20]. The complexity of the nutritional interactions between hosts and pathogens is made substantially greater by the fact that animals play host not only to invading pathogens, but also to entire communities of commensal and symbiotic microorganisms that receive their nutrition from the host and in turn contribute essential nutrients and play a role in immune defence [21]–[24]. Gut microbiota have been shown to have profound and unanticipated effects on immune defence and inflammatory responses [23], [25]–[27], and in mammals, disturbances of the gut microbiota have been implicated in diseases such as obesity, type 1 diabetes, and various cancers [21], [23], [28]. Furthermore, diet has a strong effect on the gut microbiota [29]–[32], both by serving as a vector for microorganisms and by affecting the physical, chemical, and structural properties of the gut [33]–[37].

## The Immune System Has Multiple Components That React Differently to Nutrients

**Figure 1:**
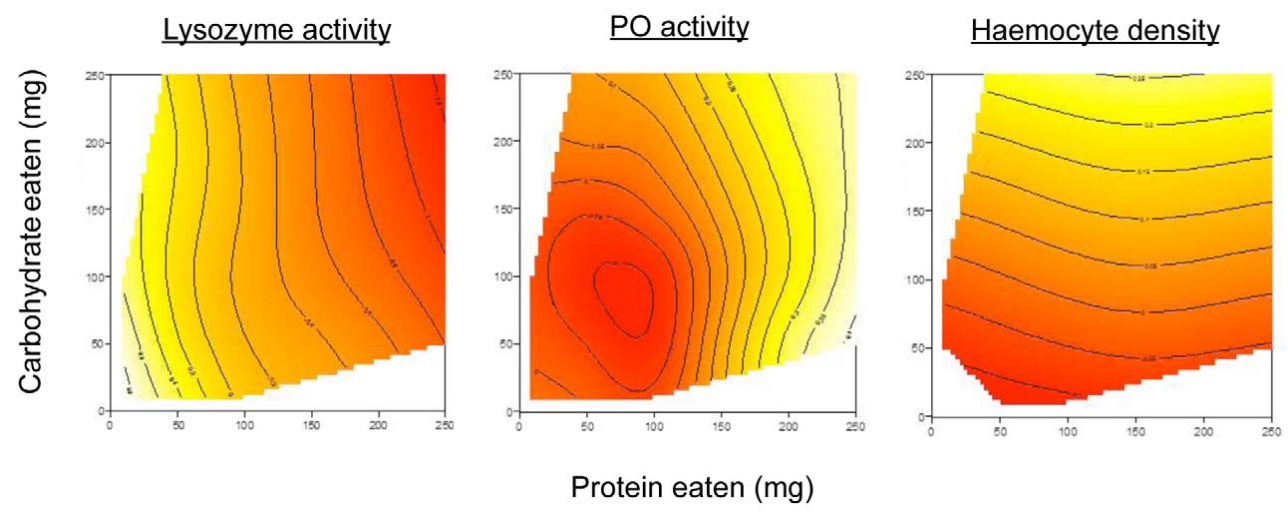
Response landscapes for three immune traits in caterpillars fed one of 20 diets differing in the ratios and amounts of protein and carbohydrate. Adapted after Cotter et al. [40].

Immune loci are the most gene-dense regions of the genome in vertebrates (e.g., [38]), and even in insects, which lack an adaptive immune response and rely solely on the innate immune system, there are abundant components to the immune response, each designed to meet particular types of immune challenge [39]. It has recently been discovered using GF designs that immune components respond differently to host nutritional state. Cotter et al. [40] restricted control and immune-challenged caterpillars to one of 20 diets varying in both the quantity and ratio of P and C. Statistical analysis of response surfaces (Figure 1) showed that immune traits are differentially affected by macronutrient intake and that no diet can simultaneously optimize all components of the immune system (see below). Variation in these different traits has been shown to be repeatable and heritable [41], [42] and linked to functional immune outcomes [43], [44]. This raises the intriguing prospect that an animal might adjust its food selection to support immune components that best resist a given infection and perhaps also support a healthy microbial community.

## A Framework to Address the Complexity of Nutritional Immunology

**Figure 2:**
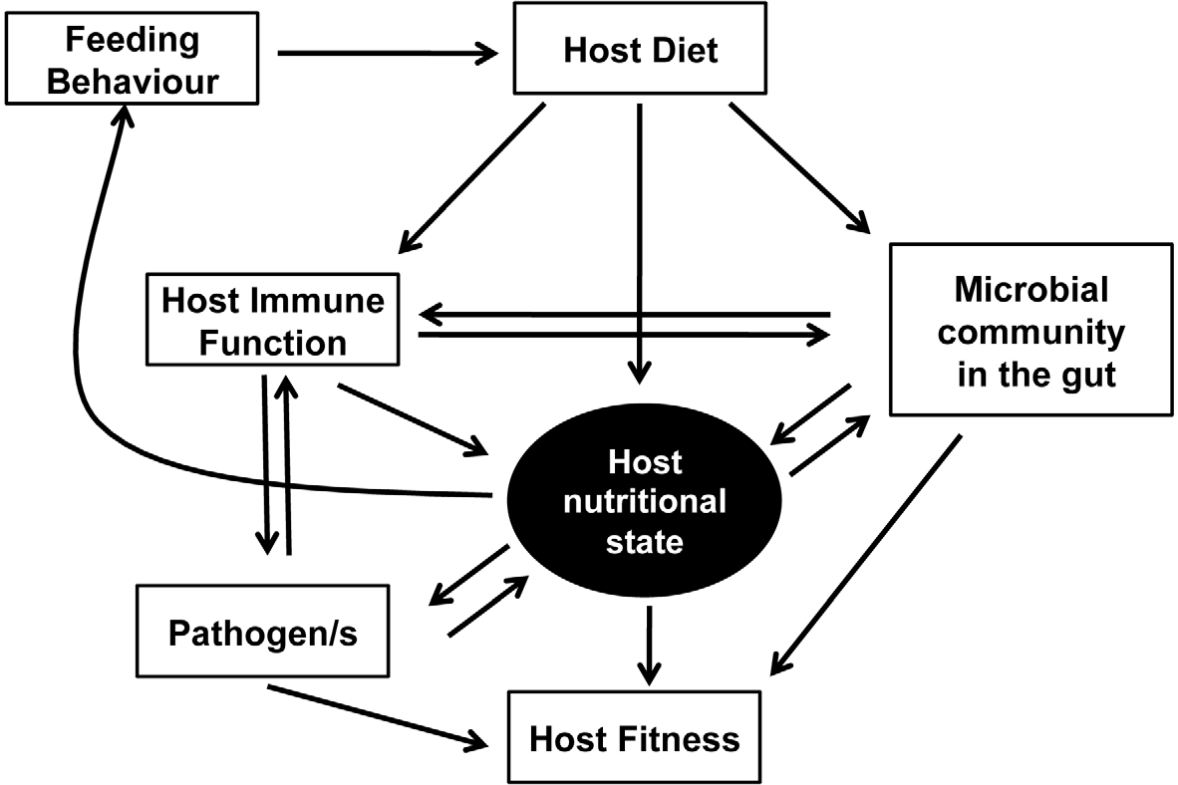
The network of interactions between nutrition and immunity. Diet affects host nutritional state and immune status, both of which interact with microbial symbionts, commensals, and pathogens to affect the fitness of all partners. Because nutrient feedbacks modulate host feeding behaviour, the potential exists for the host to adjust its diet to optimise its microbial interactions and increase resistance to infection. Alternatively, parasites and pathogens might subvert host feeding behaviour to their nutritional advantage.

When the above mentioned complexities are considered, it becomes clear that an understanding of nutritional immunology must take account of a web of interactions between components. These include the nutritional quality of the diet, host feeding behaviour, host nutritional state, the growth of pathogen populations, the host-associated microbial community, multiple measures of host immune function and, ultimately, evolutionary considerations such as host and microbial fitness and selection processes (Figure 2).

When exploring this network of interactions, the first step (primary manipulation) is to define the effects of nutrition on the network. The recent study of Lee et al. [45] on *Drosophila* offers an example of how GF designs might be used in such an analysis of nutritional immunology. In that study, systematically varying the protein and carbohydrate content allowed variables including host lifespan and lifetime egg production to be mapped as response surfaces onto nutrient intake arrays, thereby parsing the consequences of nutritional state on these key life-history traits and providing a baseline for detailed physiological and molecular analysis. Using the same technique, Cotter et al. [40] (see above) mapped several immune traits onto P-C intake arrays (Figure 1), providing evidence that immune components responded in a nutrient-specific manner. Whether these different responses were driven by differing nutritional demands of the various immune traits, direct effects of nutrition on patterns of immune gene expression, or an indirect effect of changes to microbial communities in the gut or elsewhere in the body, remains to be discovered.

A more complete study of nutritional immunology would require including response surfaces for gut and body microbial communities, as well as a more detailed assessment of immune pathways, e.g., IMD and Toll antimicrobial peptide pathways. Having quantified the effects of diet composition, the responses of the network to perturbations could then be measured. This could be done by inoculating hosts with pathogens that challenge different components of the innate immune system; by using host strains deficient in different components of the immune response; using RNAi to knockdown particular immune genes; or by manipulating the commensal microbiota through antibiotic treatment. Finally, hosts could be offered the opportunity to express nutritional self-medication in experimental designs in which they are offered a choice of nutritionally complementary foods [45]. Studying the individual components of this complex interaction will allow us to formulate null models against which specific hypotheses can be formulated and tested.

Considering the complex nature of nutritional immunology, we argue that a description of the network of interactions that define the relationships between nutrition, immune function, infection, and microbiota is essential to provide a more comprehensive and robust understanding of the key determinants of the outcome of host–pathogen interactions. The GF provides a powerful organising framework for achieving such a synthesis.
